# Chemical and Medical Qualities of the Apocynum Cannabinum

**Published:** 1833

**Authors:** 


### II. ANALYTICAL.

1. *Chemical and Medicinal Qualities of the Apocynum Cannabinum.*—Professor Griscom, of New-York, in a well written paper, inserted in the 33d No. of the American Journal, has presented to the profession as a new and valuable medicine, the *Apocynum Cannabinum*, or Indian hemp, one of our common plants. The bark of the root is most abundant in active matter, which is chiefly soluble in water. It may be administered in decoction, in tincture, or in substance, but he prefers the first mode.

"The Indian hemp, when taken internally, appears to have four different and distinct operations upon the system:—1st. As an emetic. 2d. As a purgative. 3d. As a sudorific. 4th. As a diuretic. Each of these effects it produces almost invariably. Its first operation, when taken into the stomach is that of producing nausea, if given in sufficient quantity, (which need not be large,) and if this is increased, vomiting will be the result. It very soon evinces its action upon the peristaltic motions of the prima viæ, by producing copious feculent and watery discharges, particularly the latter, which action, when once excited, is very easily continued, by the occasional administration of a wine-glassful of the decoction. The next operation of this remedy is upon the skin, where it displays its sudorific properties often in a very remarkable manner. Copious perspiration almost invariably follows its exhibition, to which effect is, in a great measure, attributed by some, the powerful influence it exercises over the various forms of dropsy. The activity of its diuretic properties does not appear to be so great in many instances as in others. In the first three or four cases related, the urinary secretion, although somewhat increased in quantity, was not such as to be commensurate with the effect produced upon the disease by the exhibition of the medicine. In other instances its diuretic operation has been more manifest, causing very profuse discharges of urine, and in a very short time relieving the overloaded tissues of their burden.

Dropsy is, I believe, the only morbid affection for the relief of which the powers of this plant have been brought into successful requisition. Its very active, and often violent operation would seem, in a great measure to preclude its use in diseases which are accompanied with much febrile excitement. Yet one might readily suppose that some of its particular properties might be very advantageously sought for in some diseases where much arterial excitement was not present—especially its emetic, sudorific, or cathartic properties, each of which operations might probably be separately obtained by giving it in well regulated doses."

